# Trajectory Tracking and Obstacle Avoidance of Robotic Fish Based on Nonlinear Model Predictive Control

**DOI:** 10.3390/biomimetics8070529

**Published:** 2023-11-06

**Authors:** Ruilong Wang, Ming Wang, Yiyang Zhang, Qianchuan Zhao, Xuehan Zheng, He Gao

**Affiliations:** 1 School of Information and Electrical Engineering, Shandong Jianzhu University, Jinan 250101, China; 2 Department of Automation, Tsinghua University, Beijing 100018, China; 3 Shandong Zhengchen Technology Co., Ltd., Jinan 250101, China

**Keywords:** robotic fish, NMPC, trajectory tracking, obstacle avoidance, DWA

## Abstract

The attainment of accurate motion control for robotic fish inside intricate underwater environments continues to be a substantial obstacle within the realm of underwater robotics. This paper presents a proposed algorithm for trajectory tracking and obstacle avoidance planning in robotic fish, utilizing nonlinear model predictive control (NMPC). This methodology facilitates the implementation of optimization-based control in real-time, utilizing the present state and environmental data to effectively regulate the movements of the robotic fish with a high degree of agility. To begin with, a dynamic model of the robotic fish, incorporating accelerations, is formulated inside the framework of the world coordinate system. The last step involves providing a detailed explanation of the NMPC algorithm and developing obstacle avoidance and objective functions for the fish in water. This will enable the design of an NMPC controller that incorporates control restrictions. In order to assess the efficacy of the proposed approach, a comparative analysis is conducted between the NMPC algorithm and the pure pursuit (PP) algorithm in terms of trajectory tracking. This comparison serves to affirm the accuracy of the NMPC algorithm in effectively tracking trajectories. Moreover, a comparative analysis between the NMPC algorithm and the dynamic window approach (DWA) method in the context of obstacle avoidance planning highlights the superior resilience of the NMPC algorithm in this domain. The proposed strategy, which utilizes NMPC, demonstrates a viable alternative for achieving precise trajectory tracking and efficient obstacle avoidance planning in the context of robotic fish motion control within intricate surroundings. This method exhibits considerable potential for practical implementation and future application.

## 1. Introduction

The utilization of ocean and river resources by humans has led to the growing prominence of underwater robots in various applications. Underwater robots are utilized in several sectors, including civil domains such as marine environmental monitoring, maritime search and rescue, seabed resource exploration, and aquatic biology research. Additionally, they find applications in military sectors for reconnaissance activities and the tracking of enemy submarines [1,2]. Research on trajectory tracking and obstacle avoidance planning for underwater robots is considered a prominent area of exploration within the realm of marine technology.

Robotic fish, classified as a subset of underwater robots, possess attributes that align with those of a time-variant, strongly coupled, and multi-input multi-output nonlinear system. Trajectory tracking control and obstacle avoidance planning control have garnered substantial attention from scholars both locally and internationally due to their prospective uses [3,4]. In the realm of underwater robotics, it is common to observe disparities between the trajectory followed by these machines and the intended reference trajectory. Consequently, the ability to safely navigate around obstacles is frequently compromised when such situations arise. The primary cause of this issue can be traced to the utilization of non-standard mathematical models by underwater robots and the implementation of poor control mechanisms. Extensive study has been performed by researchers worldwide to address the issues encountered by underwater robot systems in trajectory tracking and obstacle avoidance planning, with the aim of improving the precision and dependability of these features. Several effective control approaches have been presented to tackle these difficulties.

Up to this point, the predominant techniques employed for trajectory tracking of underwater robots consist of traditional proportional integral derivative (PID) control [5,6], sliding mode control, fuzzy control [7,8], neural network control [9,10], intelligent control [11,12], and the pure pursuit (PP) method. The PID method, being a highly prevalent control strategy, has been extensively employed in the domain of underwater robot trajectory tracking. The trajectory tracking control can utilize a neural network-based PID system, aiming to overcome challenges posed by external disturbances, such as wind and water currents, which hinder the construction of accurate dynamic models for underwater robots [13]. This approach enables real-time tracking of desired heading angles and target paths. In order to mitigate the impact of external disturbances on the variation of control coefficients in underwater robots, a series of nonlinear PID controller designs for trajectory tracking can be employed, utilizing saturation functions [14]. The sliding mode control technique, which is widely recognized for its robust capabilities, is frequently employed in the context of dynamic tracking control for underwater robots. Luo et al. [15] presented a control strategy that utilizes the backstepping method and sliding mode control technique to effectively address concerns related to horizontal plane trajectory tracking. This approach allows for the quick tracking of target trajectories while ensuring sustained tracking efficacy. Underwater robots utilize a control technique for fixed-time trajectory tracking, effectively addressing the chattering problem generated by sliding mode control gains [16]. In the field of fuzzy control methods, a variable fuzzy-based predictive controller framework has been introduced. This framework is designed to address the challenge of dynamic three-dimensional underwater trajectory tracking for autonomous underwater robots [17]. The adjustability of the structure and settings of this controller can be performed in real-time through online means. Zhu et al. [18] addressed the issue of actuator saturation in the context of trajectory tracking for underwater robots. To mitigate this problem, Zhu proposed a control solution that combines fuzzy logic and backstepping techniques. This approach allows for regulated variations in velocity that fall within acceptable ranges, as indicated by reference. Within the framework of neural network control methods, the underwater robot controller can autonomously track targets in the presence of uncertain dynamics and seawater interference. This controller utilizes radial basis functions and nonsingular terminal sliding mode control technology, achieving asymptotic stability of the target tracking controller as demonstrated through the application of Lyapunov stability principles [19]. Underwater robots enhance their autonomous maneuvering capabilities for trajectory tracking tasks in perturbed underwater environments by employing a dynamic neural control system and adaptive neural controllers [20]. Within the domain of PP approaches, Sun et al. [21] examined the precision concerns associated with conventional PP algorithms and put forth an enhanced tracking algorithm. The design in question entails the adaptive modification of the preview distance, which is determined by the steering angles and deviation angles. The PP algorithm adjusts lateral deviation by selecting different targets and dynamically modifying the preview distance, addressing the issue of subpar tracking performance resulting from inadequate selection of the preview distance [22]. The existing methods for trajectory tracking have undergone significant advancements, resulting in highly refined methodologies that provide meticulous monitoring by leveraging exact robot modeling. Nevertheless, the current body of research predominantly focuses on the development of kinematic controllers for ground-based robots, specifically designed to navigate basic and conventional smooth courses. The authors neglect to take into account the intricate nature of underwater settings and how they can impact the movement of robots. As a result, the controllers they have devised are not suitable for supporting the navigation of underwater robots through complex or sudden turns, which could potentially cause deviations from the intended trajectory.

In recent years, there has been significant progress in the development of autonomous obstacle avoidance technology in the field of underwater robotics. This has led to an increase in research efforts by both local and international research teams, focusing on the study of autonomous obstacle avoidance planning for underwater robots. In order to guarantee the autonomous ability of underwater robots to avoid obstacles in scenarios including multiple stationary objects, Li introduced a control approach for obstacle avoidance in a spherical underwater robot. This strategy relies on the utilization of an ultrasonic sensor array. This approach considers the kinematic and dynamic models of the robot as well as the properties of ultrasonic sensors [23]. In order to address the nonlinear and fully constrained tracking as well as obstacle avoidance issues, mobile robots utilize an extended state observer to estimate unknown disturbances. Additionally, the robot effectively resolves obstacle avoidance problems during movement by employing obstacle region distance cost potential functions [24]. To address challenges related to collision avoidance, robots can utilize a framework of motion planning and tracking control. This framework employs a multi-constraint numerical optimization technique for motion planning, enabling effective control for obstacle avoidance [25]. Xiang et al. [26] presented an enhanced obstacle avoidance approach for mobile robots, specifically focusing on the obstacle avoidance problem. This method builds upon the Dynamic Window Approach (DWA) and was proposed by Xiang. This method integrates fuzzy logic to dynamically modify weight coefficients in real-time, addressing the limited adaptability observed in the conventional DWA methodology. This is achieved through the analysis of target and obstacle information. Liu made enhancements to the conventional DWA algorithm, which is commonly employed in path planning studies for land-based robots, in order to address the unique challenges posed by underwater environments. These challenges include the need to attain globally optimal paths and perform real-time dynamic obstacle avoidance. This improvement was documented in a research paper [27]. Conventional obstacle avoidance methods commonly employ obstacle information to adjust control variables for avoidance or produce avoidance paths. Nevertheless, these methods do have certain limitations. The utilization of obstacle data to adjust control parameters for the purpose of avoiding obstacles has the potential to cause disorganized movement of the robot, leading to instability in control due to substantial changes in both linear and rotational velocities.

The conventional control techniques employed in the field of robotic fish frequently depend on PID control or empirical methodologies. However, these methods demonstrate some shortcomings in terms of their performance and ability to withstand challenges in intricate underwater settings. The present work introduces several novel contributions, which are outlined below:

(1) The utilization of nonlinear model predictive control (NMPC) in the realm of motion control for nonlinear robotic fish systems. This approach utilizes real-time optimization solutions to boost the adaptability of robotic fish in different surroundings, allowing for accurate trajectory tracking and obstacle navigation. In contrast to conventional trajectory planning techniques that predominantly focus on position and velocity alterations, this methodology incorporates enhanced control over acceleration.
(2) The implementation of trajectory planning and optimization algorithms based on acceleration, which enables the achievement of smoother motion for robotic fish and the reduction of excessive inertial forces during movement. Consequently, this approach improves the precision of trajectory monitoring as well as the level of comfort experienced.

The remainder of this paper is organized as follows. Section 2 presents the theoretical framework of the bionic robotic fish, encompassing key variables such as the position, velocity, and acceleration of the device. This model serves as a foundation for the implementation of motion control strategies and obstacle avoidance mechanisms. In Section 3, a controller based on NMPC is introduced. This controller formulates trajectory tracking and obstacle avoidance planning as an optimization problem. In Section 4, simulation studies are conducted to validate the efficacy of the proposed NMPC technique in the context of trajectory tracking and obstacle avoidance tasks for underwater robotic fish. The final section provides a summary of the entire article and offers insights into the potential practical implications of employing the nonlinear model predictive control method.

## 2. Modeling of Robotic Fish

**Figure 1:**
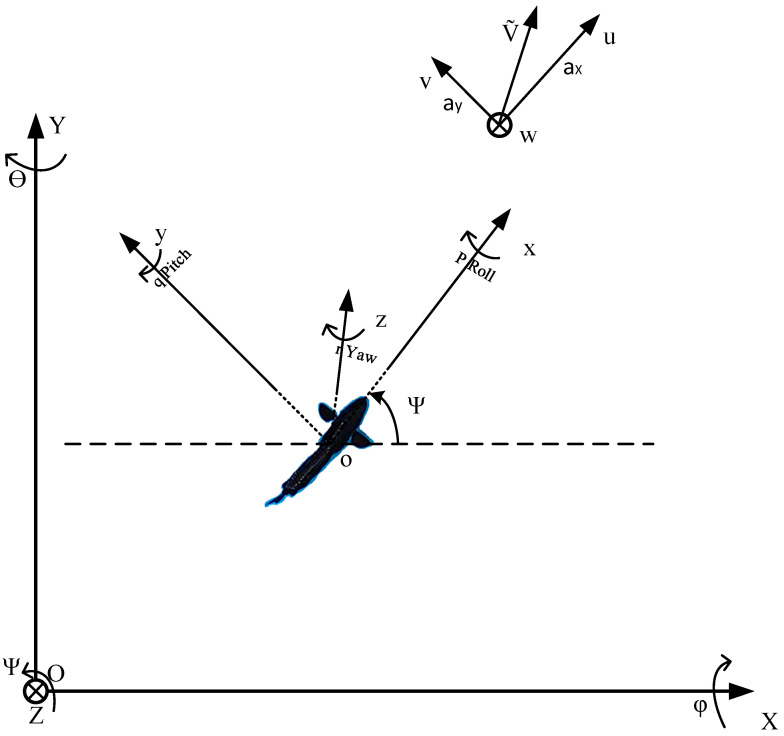
Motion coordinate system of bionic robot fish.

In order to accomplish trajectory tracking of the robotic fish, a kinematic model is created to characterize its motion, incorporating variables such as velocity, location, and rotational angles. To achieve a more sophisticated formulation of the kinematic model for the biomimetic robotic fish, it is necessary to build a coordinate system that effectively characterizes the fish’s motion. As depicted in Figure 1, the o-xyz coordinate system is defined as the carrier coordinate system of the biomimetic robotic fish, where o represents the fish’s center of mass, and the x and y axes denote the lateral and longitudinal directions of the fish, respectively, while the z-axis is oriented upward perpendicular to the fish’s body. Additionally, the O-XYZ coordinate system, also referred to as the global coordinate system or the world coordinate system, is defined. Here, the XOY plane lies parallel to the horizontal plane, and the OZ axis is oriented vertically upward [28].

The coordinates of the fish body’s center of mass in the world coordinate system are as follows: $P_{w}={\left(X,\;Y,\;Z\right)}^{T}$. The rotational vector representation of the fish body’s center of mass in the world coordinate system is expressed as follows: $A_{w}={\left(\phi ,\;\theta ,\;\psi \right)}^{T}$. The transformation of the center of mass of the fish body in the carrier coordinate system to the world coordinate system can be achieved by utilizing a rotational vector. As a result, the matrix that represents the transformation of the linear velocity of the fish body’s center of mass from the carrier coordinate system to the world coordinate system can be mathematically described in the following manner

(1) Rbw=R(ψ)R(θ)R(ϕ)

where

$$R(\psi )=\left[\begin{array}{ccc} C_{\psi } & -S_{\psi } & 0\\ S_{\psi } & C_{\psi } & 0\\ 0 & 0 & 1 \end{array}\right],\;R(\psi )=\left[\begin{array}{ccc} C_{\theta } & 0 & S_{\theta }\\ 0 & 1 & 0\\ -S_{\theta } & 0 & S_{\theta } \end{array}\right],\;R(\psi )=\left[\begin{array}{ccc} 1 & 0 & 0\\ 0 & C_{\phi } & -S_{\phi }\\ 0 & S_{\phi } & C_{\phi } \end{array}\right],$$

(2) Rbw=CθCψ−CϕSψ+CϕCθCψSϕSψ+CϕSθCψCθSψCϕCψ+SϕSθSψ−SϕCψ+CϕSθCψ−SθSϕCθCϕCθ.

In a similar manner, the matrix that represents the transition of the angular velocity of the fish body’s center of mass from the coordinate system of the carrier to the coordinate system of the world can be expressed as follows.

(3) Tbw=1SϕTθCϕTθ0Cϕ−Sϕ0Sϕ/CθCϕ/Cθ

Here, $S_{x}$ represents sin(x), $C_{x}$ represents cos(x), and $T_{x}$ represents tan(x); it is also evident that R(x) is an orthogonal matrix, satisfying the relation $R^{-1}(x)=R^{T}(x)$ [29].

When $V_{b}={\left(u,\;v,\;w\right)}^{T}$ represents the linear velocity components of the robotic fish along the x, y, and z axes in the carrier coordinate system, and $\Omega_{b}={\left(p,\;q,\;r\right)}^{T}$ denotes the angular velocity components of the fish’s center of mass in the same coordinate system, the position and orientation of the fish’s center of mass in the world coordinate system can be mathematically represented using iterative formulas.

(4) $$\left[\begin{array}{c} P_{w}\\ A_{w} \end{array}\right]=\int \left[\begin{array}{c} {\dot{P}}_{w}\\ {\dot{A}}_{w} \end{array}\right]dt$$

The iterative formula for the velocity of the fish’s center of mass is as follows.

(5) $$\left[\begin{array}{c} V_{b}\\ \Omega_{b} \end{array}\right]=\int \left[\begin{array}{c} {\dot{V}}_{b}\\ {\dot{\Omega }}_{b} \end{array}\right]dt$$

where ${\dot{V}}_{b}$ represents the linear acceleration components of the fish’s center of mass in the carrier coordinate axes, while ${\dot{\Omega }}_{b}$ signifies the angular acceleration components in the carrier coordinate system. The kinematic equation of the robotic fish can be formulated as depicted in Equation (7).

(6) P˙wA˙w=Rbw03×303×3TbwVbΩb

(7) $$\left\{\begin{array}{l} \dot{X}=uC_{\theta }C_{\psi }+v(-C_{\phi }S_{\psi }+C_{\phi }C_{\theta }C_{\psi })+w(S_{\phi }S_{\psi }+C_{\phi }S_{\theta }C_{\psi })\\ \dot{Y}=uC_{\theta }S_{\psi }+v(C_{\phi }C_{\psi }+S_{\phi }S_{\theta }S_{\psi })+w(-S_{\phi }C_{\psi }+C_{\phi }S_{\theta }C_{\psi })\\ \dot{Z}=-uS_{\theta }+vS_{\phi }C_{\theta }+wC_{\phi }C_{\theta }\\ \dot{\phi }=p+qS_{\phi }T_{\theta }+rC_{\phi }T_{\theta }\\ \dot{\theta }=qC_{\phi }-rS_{\phi }\\ \dot{\psi }=qS_{\phi }/C_{\theta }+rC_{\phi }/C_{\theta } \end{array}\right.$$

Here, only the planar motion of the robotic fish is taken into consideration. This implies that substituting θ=ϕ=0, p=q=0, w=0 into (7) yields the following result.

(8) $$\left\{\begin{array}{l} \dot{X}=u\cos\psi -v\sin\psi \\ \dot{Y}=u\sin\psi +v\cos\psi \\ \dot{\psi }=r \end{array}\right.$$

The kinematic model of the robotic fish pertains to the temporal progression of its motion states, which often includes details such as position, velocity, and orientation. Nevertheless, in order to incorporate the acceleration of the fish, it becomes imperative to transition from the kinematic model to a dynamic model. The dynamic model takes into account not just variations in velocity and position, but also integrates the impact of acceleration on these variables.

The dynamic model of the robotic fish exhibits a strong correlation between acceleration and steering angle. The rate of change in acceleration has a direct influence on variations in the steering angle, while modifications in the steering angle, in turn, have a reciprocal effect on both the direction and magnitude of acceleration. The mathematical representation of the relationship between acceleration and steering angle can be expressed as follows

(9) $$\left\{\begin{array}{l} \ddot{x}=a_{x}\\ \ddot{y}=a_{y}\\ \dot{\psi }=\frac{a_{y}}{u}-\frac{a_{x}}{v} \end{array}\right.$$

where $a_{x}$ and $a_{y}$ denote the accelerations along the lateral and longitudinal axes of the robotic fish, respectively. The dynamic model of the robotic fish is presented as

(10) $$\left\{\begin{array}{l} \ddot{x}=a_{x}\\ \ddot{y}=a_{y}\\ \dot{\psi }=\frac{a_{y}}{u}-\frac{a_{x}}{v}\\ \dot{X}=u\cos\psi -v\sin\psi \\ \dot{Y}=u\sin\psi +v\cos\psi \end{array}\right.$$

This study enhances the kinematic model by incorporating acceleration, so transforming it into a dynamic model. This modification allows for a more accurate representation of the motion behavior exhibited by the robotic fish. The inclusion of acceleration in the analysis facilitates a more comprehensive comprehension of the dynamic properties shown by the fish. This holds special significance in the context of designing control algorithms and optimizing the motion performance of the fish.

## 3. A NMPC-Based Control Method for Robotic Fish

### 3.1. Overview of NMPC Algorithm

For a nonlinear system, consider the following general form of a discrete model

(11) $$\begin{array}{l} \xi (t+1)=f(\xi (t),u(t))\\ \xi (t)\in \chi ,\;u(t)\in \Gamma \end{array}$$

where f() represents the state transition function of the system, ξ denotes an $n_{s}$-dimensional state variable, u signifies an $m_{c}$-dimensional control variable, χ stands for state variable constraints, and Γ refers to control variable constraints.

Setting f(0, 0)=0 as a stable point of the system, which also serves as the control objective, for any given time domain N, the following optimization objective function $J_{N}()$ is considered

(12) $$J_{N}(\xi (t),U(t))=\sum_{k=t}^{t+N-1}l(\xi (k),u(k))+P(\xi (t+N))$$

where $U(t)={\left[u(t),\cdot \cdot \cdot ,u(t+N-1)\right]}^{T}$ represents the control input sequence within the time domain N, and ξ(t) signifies the trajectory of the system’s state vector under the influence of the input vector sequence U(t). The first term l() in the optimization objective function represents the tracking capability towards the desired output, and the second term P() signifies terminal constraints [30].

At any arbitrary time, the predictive model of the NMPC system can forecast all state variables of the system from time t+1,t+2 to ${t+N}_{p}$, where $N_{p}$ represents the prediction horizon, based on the actual outputs and control inputs of the biomimetic robotic fish up to the current time. NMPC then, in accordance with the objective function and system constraints defined for the specific system, computes all control inputs from time t to $t+N_{c}$, where $N_{c}$ signifies the control horizon [31].

NMPC aims to solve, at each time step, the following constrained finite-horizon optimization problem

(13) $$\underset{U_{t},\xi_{t+1,t},\cdots \xi_{t+N,t}}{\min}\;J_{N}(\xi_{t},U_{t})$$

(14) $$s.t.\;\xi_{k+1,t}=f(\xi_{k,t},u_{k,t}),\;k=t,\cdots ,t+N-1$$

(15) $$\xi_{k,t}\in \chi \;k=t+1,\cdots ,t+N-1$$

(16) $$u_{k,t}\in \Gamma \;k=t,\cdots ,t+N-1$$

(17) $$\xi_{t,t}=\xi (t)$$

(18) $$\xi_{N,t}\in \chi_{fin}$$

where Equation (14) represents the state constraints imposed by the system, Equations (15) and (16) denote the constraints on the state vector and control input vector, respectively, Equation (17) entails the initial state constraint, and Equation (18) pertains to the terminal state constraint.

**Figure 2:**
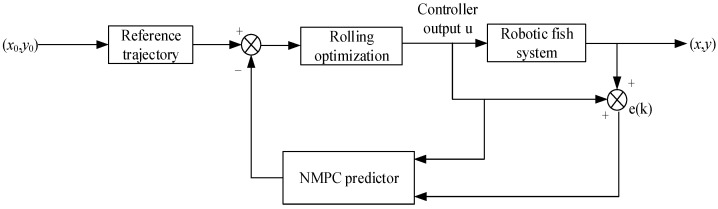
NMPC control flowchart.

The framework flowchart of NMPC control is shown in Figure 2.

Here, $\left(x_{o},y_{o}\right)$ represents the initial position of the bionic robotic fish at the current moment, e(k) denotes the error between the predicted pose and the reference trajectory, and (x,y) signifies the position of the bionic robotic fish in the next moment after prediction.

**Figure 3:**
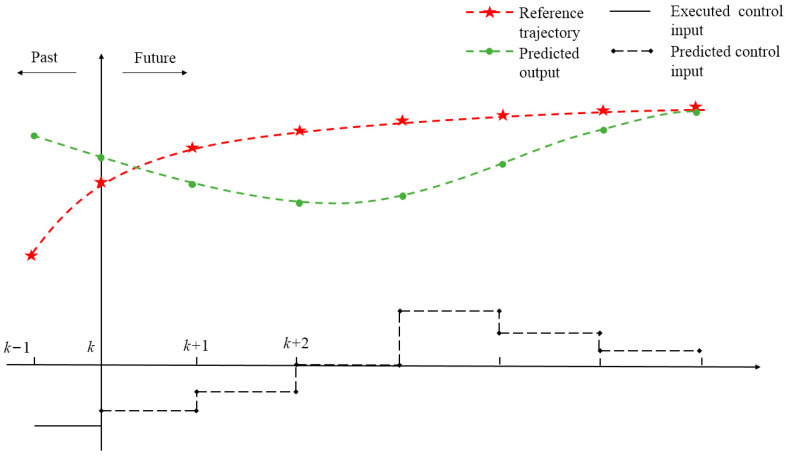
Nonlinear Model Predictive Control for Biomimetic Robotic Fish.

Figure 3 depicts the predictive control framework employed for the nonlinear model of the bionic robotic fish.

Figure 3 illustrates the representation of several components in the context of a robotic fish. The red dashed line corresponds to the reference trajectory, while the green dashed line represents the predictive output of the robotic fish for the reference trajectory. Additionally, the black dashed line denotes the control input created by the predictive model. At any time step k, the predictive model of the NMPC system can predict all state variables from time step k+1 to $k+N_{p}$, where $N_{p}$ is the prediction horizon, based on the actual output and control input of the robotic fish in the subsequent time steps starting from the current time step k. NMPC computes the control inputs for all time steps from k to $k+N_{c}-1$, where $N_{c}$ is the control horizon, according to the objective function and system constraints defined for the specific system.

Finally, the control inputs obtained from the optimized control sequence at time step k are applied to the controlled system. The process is repeated at time step k+1 to obtain the next set of control inputs. This rolling optimization continues until the predictive output of the robotic fish converges to the reference trajectory, completing the NMPC predictive control.

### 3.2. Functions for Obstacle Avoidance and Objective Functions

The obstacle avoidance function entails modifying the penalty function value by considering the discrepancy between the distance of obstacles and the target point. In this case, a greater function value is assigned to a closer distance. An augmentation in the weight coefficient has a tendency to render the planning outcomes more cautious. In scenarios when the robotic fish lacks information regarding obstacles, the weight coefficient will have no impact on the planning results. When encountering substantial measurement or estimation mistakes in the state of the robotic fish, it is possible to utilize higher weight coefficients. Nevertheless, this phenomenon also results in a rise in tracking deviation, as demonstrated in the subsequent analysis

(19) $$J_{obs,i}=\frac{S_{obs}v_{i}}{{\left(X_{i}-x_{o}\right)}^{2}+{\left(Y_{i}-y_{o}\right)}^{2}+\zeta }$$

where $S_{obs}$ is the weight coefficient, $v_{i}=v_{x}^{2}+v_{y}^{2}$ is the square of the robotic fish’s swimming velocity, $\left(X_{i},Y_{i}\right)$ represents the position coordinates of the obstacle point in the world coordinate system, $\left(x_{o},y_{o}\right)$ denotes the coordinates of the robotic fish’s center of mass in the world coordinate system, and ζ is a very small positive value introduced to prevent division by zero situations in the denominator.

The objective function at the trajectory planning level is to minimize deviations from the global reference path and achieve obstacle avoidance. The act of circumventing hurdles is achieved by implementing penalty functions in the following manner

(20) $$\begin{array}{l} \min\sum_{i=1}^{N_{p}}{\left\|\eta_{pre}(t+i\;|\;t)-\eta_{ref}(t+i\;|\;t)\right\|}^{2}\times Q+{\left\|U_{i}\right\|}^{2}\times R+J_{obs,i}\\ s.t.\;U_{\min}\leq U_{t}\leq U_{\max} \end{array}$$

where $\eta_{pre}$ and $\eta_{ref}$ are the predicted trajectory and the reference trajectory, respectively, and Q and R are weight matrices.

The management of motion kinematic trajectory tracking and obstacle avoidance in robotic fish include the manipulation of the fish’s lateral and longitudinal accelerations to regulate its forward and backward velocity, lateral velocity, and yaw rate. This action is undertaken with the purpose of ensuring that the center of mass of the fish follows a specified reference trajectory, resulting in the continuous reduction of the discrepancy between the projected trajectory and the reference trajectory until it reaches zero.

### 3.3. Design of the NMPC Controller

NMPC employs a nonlinear model to forecast forthcoming states by considering the present state and a sequence of control inputs inside the control horizon. The process at hand is evidently characterized by an iterative methodology, wherein it is necessary to establish an explicit iteration equation in order to estimate the solution of the differential equation, considering that the control sequence is unknown. In the realm of actual engineering applications, numerical methods that are frequently employed encompass the Euler method and the fourth-order Runge–Kutta algorithm. In this part, the forward Euler approach is utilized to discretize Equation (10) and transform it into a predictive model.

(21) $$\left\{\begin{array}{l} \dot{x}(i)=\dot{x}(i-1)+T\times a_{x}(i)\\ \dot{y}(i)=\dot{y}(i-1)+T\times a_{y}(i)\\ \psi (i)=\psi (i-1)+T\times [a_{y}(i)/\dot{x}(i-1)]-T\times [a_{x}(i)/\dot{y}(i-1)]\\ X(i)=X(i-1)+T\times [\dot{x}(i-1)\cos\psi (i-1)-\dot{y}(i-1)\sin\psi (i-1)]\\ Y(i)=Y(i-1)+T\times [\dot{x}(i-1)\sin\psi (i-1)+\dot{y}(i-1)\cos\psi (i-1)] \end{array}\right.$$

Taking into account the maximum hydrostatic pressure that the robotic fish can endure while swimming underwater, the following dynamic constraints are incorporated:

(22) $$\begin{array}{l} |a_{x}|<F_{p}/m\\ |a_{y}|<F_{p}/m \end{array}$$

where $F_{p}$ represents the maximum hydrostatic pressure the fish body can endure. Equations (10) and (22) can be succinctly expressed as follows

(23) $$\begin{array}{l} \dot{\xi }(t)=f(\xi (t),u(t))\\ |u(t)|<F_{p} \end{array}$$

where $\xi ={\left[\dot{x},\dot{y},\psi ,X,Y\right]}^{T}$ consists of five discrete state vectors, which are the swimming velocities of the robotic fish in the x and y directions, the heading angle, and the Cartesian coordinates of the fish, while u(t) represents the control input.

## 4. Simulation Analysis and Validation

The paper presents trajectory tracking and obstacle avoidance simulations as a means to validate the efficacy of the NMPC algorithm in the motion control of the bionic robotic fish. This study aims to evaluate and compare the tracking performance of the NMPC tracking algorithm and the Pure Pursuit (PP) algorithm on S-shaped and cloverleaf trajectories. Furthermore, this study conducts a comparative analysis between the obstacle avoidance method of NMPC and the Dynamic Window Approach (DWA) algorithm, focusing on their respective effectiveness in avoiding obstacles along circular routes. This study employs a comparative analysis to establish the precision of the NMPC algorithm in trajectory tracking and evaluate the efficacy of its obstacle avoidance planning.

### 4.1. Trajectory Tracking Control Algorithm

#### 4.1.1. Control of S-Shaped Curve Trajectory Tracking

**Figure 4:**
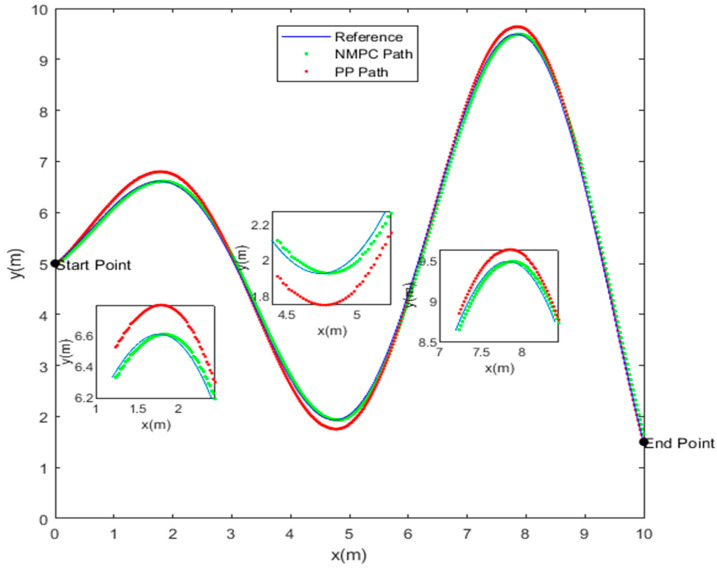
Trajectory tracking results of bionic robotic fish—S-shaped.

In a 10 m by 10 m plane, a reference trajectory is set with the starting coordinates (0, 5) and the ending coordinates (10, 1.5). Due to the computational complexity of the algorithm, a point mass model without considering size information is employed for the fish model. The initial state of the fish is denoted as $\xi ={\left[0\;0\;\pi /2\;0\;5\right]}^{T}$, and the allowable range for trajectory tracking error is 0.1 m. The NMPC controller possesses a state variable dimension of $n_{s}$ = 5, a prediction horizon of $N_{p}$ = 5, a control horizon of $N_{c}$ = 3, and a sampling time of T = 0.02 s. For the PP controller, the lookahead distance coefficient is 0.1, the lookahead distance is 1 m, and the sampling time is 0.02 s. Figure 4 presents the tracking trajectories of the fish. It can be observed that both the NMPC and PP tracking algorithms successfully achieve trajectory tracking from the starting point to the destination.

**Figure 5:**
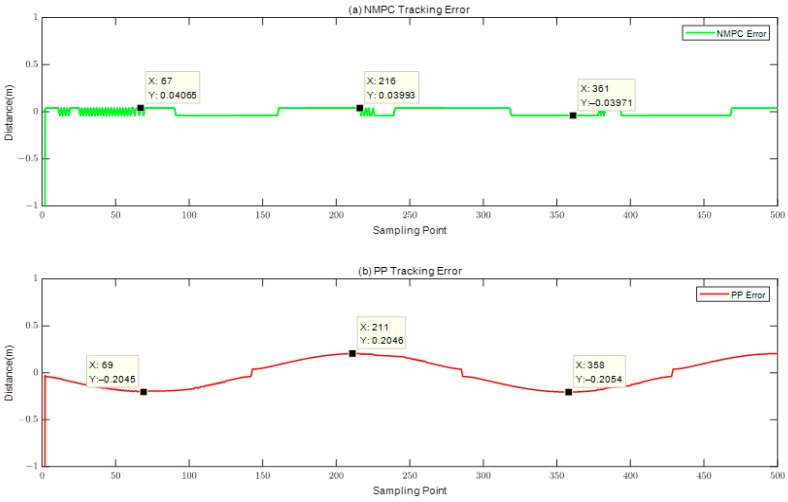
Trajectory Error Comparison of NMPC and PP.

Although both of the aforementioned algorithms are capable of achieving trajectory tracking, it is important to note that their respective tracking effects exhibit notable differences. The PP tracking method has a tendency to diverge from the reference trajectory, particularly when encountering steep curves. In contrast, the NMPC tracking approach does not demonstrate this behavior. In order to offer a more comprehensive analysis of the tracking effects exhibited by the two algorithms, a comparison was undertaken by examining the disparities between the trajectory tracked by each algorithm and the reference trajectory. The findings are displayed in Figure 5.

The superiority of the robotic fish’s tracking accuracy under the control of the NMPC algorithm, as compared to the PP algorithm, is clear, leading to reduced errors. The PP tracking algorithm exhibits notable inaccuracies at sample points 69, 211, and 358, wherein the largest tracking error amounts to 0.2046 m. The observed divergence above the acceptable threshold for tracking error is by a magnitude of 204.6%. This deviation may be attributable to abrupt changes in the trajectory occurring at these specific sample locations. In contrast, the trajectory error observed in the NMPC tracking method consistently remains below 0.1 m, while the turning errors converge within the allowed range of error. Upon comparing the error values of the two algorithms, it becomes apparent that the NMPC method demonstrates superior performance in relation to overshoot and settling time when compared to the PP algorithm. The findings of this study provide empirical evidence supporting the superior accuracy and stability of the NMPC algorithm in trajectory tracking when compared to the PP algorithm.

**Figure 6:**
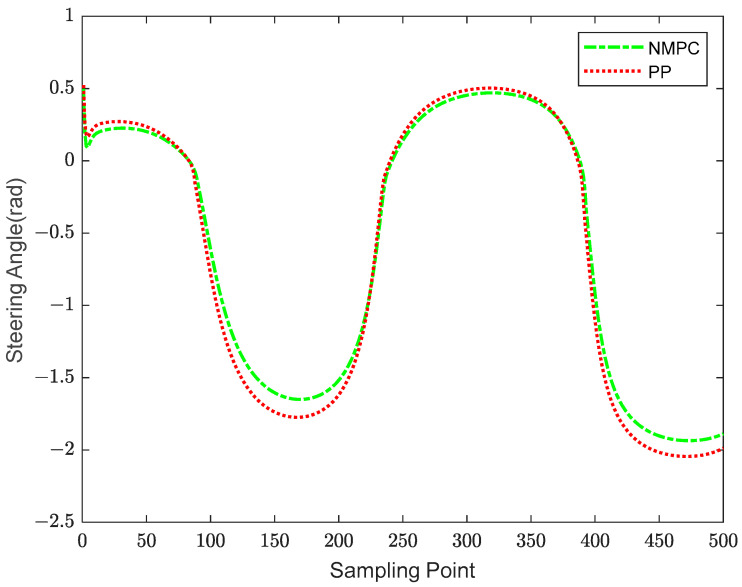
Turning angles comparison of NMPC and PP.

The occurrence of trajectory tracking errors may be attributed to the orientation angle of the robotic fish, denoting the angle between the lateral body axis of the fish and the direction of the Earth’s -axis. Figure 6 depicts a comparison of the orientation angle between PP control and NMPC.

In the range of sample points 90 to 230, it is seen that the magnitudes of the orientation angles obtained under the PP control approach tend to be greater compared to those obtained using the NMPC method. The occurrence of tracking errors may be observed in Figure 4, as indicated by the excessive orientation angles between the first and second extremum points. In a comparable manner, it can be observed that the PP control method demonstrates instances of notably greater orientation angles inside the sampling point intervals of 250 to 330 and 400 to 500, whereas the NMPC control approach does not exhibit similar occurrences.

Based on Figure 4, Figure 5 and Figure 6, it can be inferred that both the NMPC and PP tracking techniques have the ability to accurately follow the predetermined trajectory. Specifically, in the context of straight or smooth pathways, each of these tracking approaches produce comparable trajectories and provide favorable control results. However, while navigating via S-shaped or sharp turning courses, there are observable differences in the trajectories followed by the PP and NMPC methods. Specifically, the NMPC technique demonstrates a higher level of accuracy in tracking trajectories compared to the PP method. The NMPC tracking approach demonstrates the ability to precisely track the reference trajectory on linear pathways, as well as achieve convergence of tracking trajectories to the reference trajectory on curved paths.

#### 4.1.2. Trajectory Tracking Control of Cloverleaf-Shaped Curves

**Figure 7:**
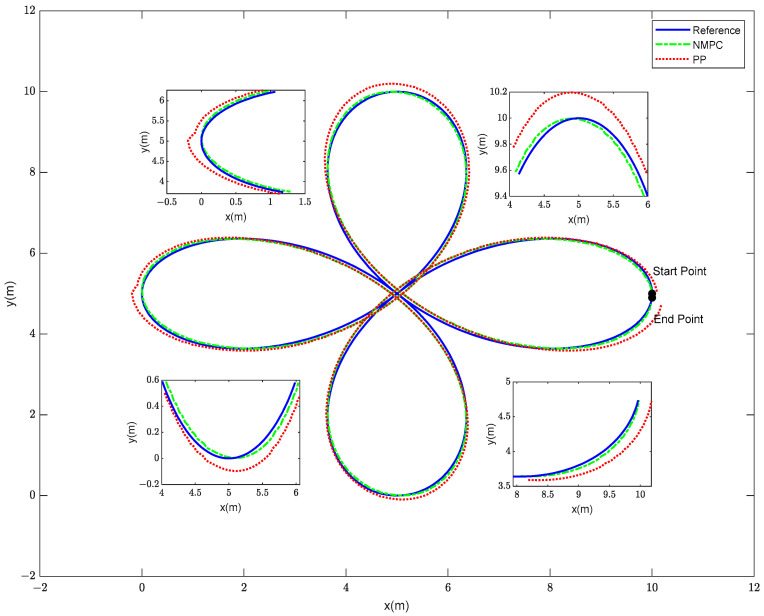
Trajectory Results of the Cloverleaf Curve for the Robotic Fish.

In order to enhance the credibility of our control method’s ability to track trajectories in a more intricate setting, subsequent to the successful tracking of the S-shaped curve trajectory, the robotic fish proceeded to navigate the more demanding cloverleaf-shaped trajectory. In contrast to the S-shaped curve, the cloverleaf curve is characterized by pronounced turns and fast fluctuations in speed, necessitating a considerable level of agility and accurate control capabilities from the robotic fish system. The initial state of the fish is denoted as $\xi ={\left[0\;0\;\pi /2\;10\;5\right]}^{T}$, and the allowable range for trajectory tracking error is 0.2 m. The NMPC controller possesses a state variable dimension of $n_{s}$ = 5, a prediction horizon of $N_{p}$ = 5, a control horizon of $N_{c}$ = 3, and a sampling time of T = 0.02 s. For the PP controller, the lookahead distance coefficient is 0.1, the lookahead distance is 1 m, and the sampling time is 0.02 s. The simulation diagram illustrating the robotic fish tracking the cloverleaf trajectory is shown in Figure 7.

**Figure 8:**
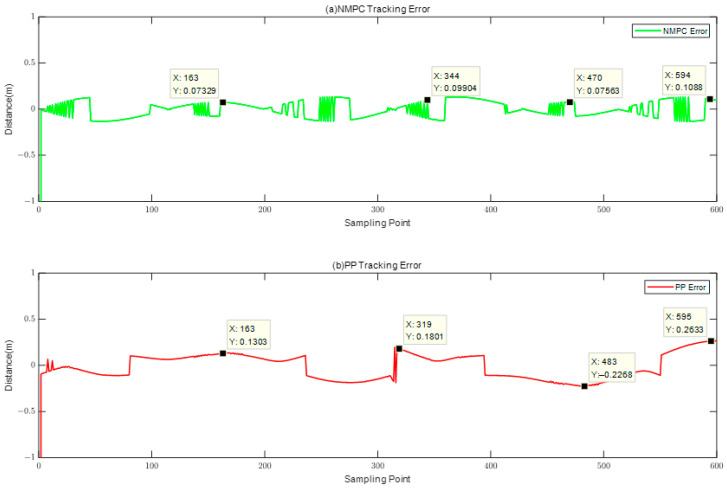
Trajectory Errors in NMPC Tracking and PP Tracking.

The provided diagram illustrates that both the NMPC and PP tracking methods are capable of achieving trajectory tracking for the cloverleaf curve. However, distinct disparities between the two approaches become apparent when examining the locally magnified image. The tracking approach employed by the PP system exhibits a tendency to execute premature turns in instances where the steering angle exceeds a certain threshold, resulting in suboptimal adherence to the intended curved trajectory. In order to provide a more comprehensive illustration of the tracking impacts of the two techniques, an analysis is conducted to determine the disparities between the tracking trajectory and the reference trajectory. The obtained findings are visually presented in Figure 8.

**Figure 9:**
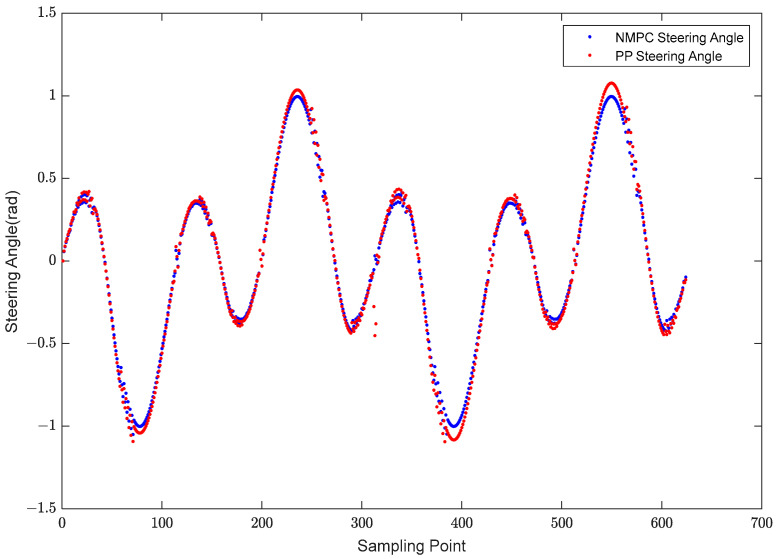
Turning Angles under NMPC Control and PP Control.

The experimental results demonstrate that by employing the NMPC method as depicted in Figure 8, the robotic fish exhibits a significantly reduced tracking error, tending towards zero. The observed tracking flaws in both approaches primarily manifest at the extremities of the cloverleaf pattern. The PP tracking method exhibits a maximum tracking error of 0.2633 m, above the acceptable range for tracking error by 131.65%. On the other hand, the NMPC tracking approach exhibits a maximum trajectory error of 0.1088 m, which falls within the acceptable limit for tracking errors. This observation confirms that the NMPC tracking approach exhibits more precision in complicated situations when compared to the PP tracking method. Figure 9 depicts a visual representation of the contrasting turning angles exhibited by the robotic fish when subjected to two different control methods, namely NMPC control and PP control.

Figure 9 illustrates that, on the whole, there exists a marginal disparity between the turning angles when subjected to NMPC control and PP control. Nevertheless, it is worth noting that the turning angle under PP control exceeds that under NMPC at the extreme points. The occurrence of large turning angles in PP control at these critical places has the potential to result in tracking errors, hence causing divergence from the intended reference trajectory.

Based on the analysis of Figure 7, Figure 8 and Figure 9, it is apparent that, within the intricate context of the four-leaf clover curve, both the NMPC and PP control approaches demonstrate effective trajectory tracking capabilities, exhibiting commendable performance in this regard. Nevertheless, there are discernible distinctions between the PP and NMPC tracking systems at the extremities of the four-leaf clover. The tracking trajectory of the PP method has a tendency to deviate from the reference trajectory at the tips, accompanied by the occurrence of anticipatory turning. In contrast, the NMPC tracking approach reveals precise convergence to the reference trajectory at the tips.

### 4.2. Validation of Obstacle Avoidance Planning and Control Algorithm

**Figure 10:**
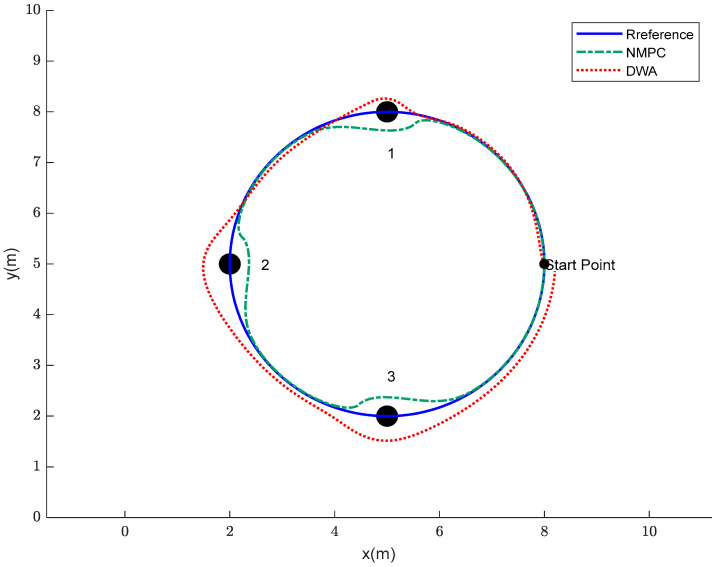
Obstacle avoidance trajectories of the robotic fish by using NMPC and DWA.

The obstacle avoidance control for the robotic fish, based on the principles of trajectory tracking, is implemented using NMPC. The process entails the identification of recently introduced obstacles and the iterative calculation of both the objective function and the obstacle avoidance function. The aforementioned iterative procedure ultimately accomplishes the objectives of tracking control and obstacle avoidance control along a predetermined trajectory. The PP algorithm does not possess inherent obstacle avoidance functionality. Its primary objective is to guide the robot along a specified course, disregarding the existence of impediments in the surrounding environment. The presence of barriers within the environment can potentially result in collisions when employing the pure PP algorithm. The DWA method has been specifically developed to address the challenges of path planning and obstacle avoidance in the context of mobile robots. The objective of this research is to facilitate the robot’s ability to promptly react, maneuver past barriers, and adhere to the predetermined path in order to achieve the desired outcome within intricate surroundings. Therefore, in the process of validating obstacle avoidance control algorithms, a comparative analysis is conducted between the NMPC obstacle avoidance algorithm and the DWA obstacle avoidance algorithm. The simulation results obtained through the utilization of NMPC and DWA obstacle avoidance algorithms on a predetermined track are depicted in Figure 10. The successful avoidance of impediments by the robotic fish is clearly demonstrated under the control of both algorithms.

In this context, the initial state of the robotic fish is defined as $\xi ={\left[0\;0\;\pi /2\;8\;5\right]}^{T}$. The NMPC controller has a state variable dimension of $n_{s}$ = 5, a prediction horizon of $N_{p}$ = 10, a control horizon of $N_{c}$ = 3, and a sampling time of T = 0.05 s. The safety margin between the robotic fish and obstacles is set at 0.3 m. The centroids of obstacles 1, 2, and 3 are located at coordinates (5, 8), (2, 5), and (5, 2) respectively, with each obstacle having a radius of 0.2 m.

**Figure 11:**
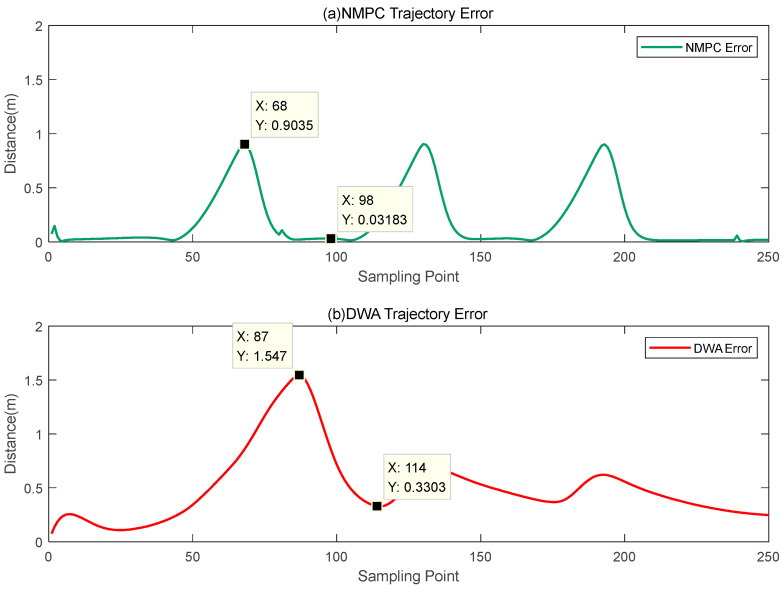
Distances between the obstacle avoidance trajectory and the reference trajectory.

Based on Figure 10, it is evident that, while both of the aforementioned approaches possess the ability to navigate around obstacles, there exists a notable discrepancy in the efficacy of their avoidance capabilities. The obstacle avoidance method known as DWA presents difficulties in terms of extended avoidance trajectories and increased avoidance radii. Furthermore, following the successful evasion of impediments, the system encounters difficulties in promptly reestablishing its predetermined direction. The obstacle avoidance system based on NMPC successfully tackles the challenge of accommodating greater radii while efficiently returning to the original trajectory following obstacle avoidance. In order to facilitate a more thorough examination of the obstacle avoidance capabilities of these two approaches, this research undertakes a calculation of the disparity between the reference trajectory and the obstacle avoidance trajectories derived from both algorithms. The findings are illustrated in Figure 11.

Upon analysis of Figure 11, it becomes evident that the DWA obstacle avoidance method demonstrates a notably extended control period compared to the NMPC obstacle avoidance method throughout the process of navigating around obstacles. Furthermore, it is seen that the avoidance trajectories generated by the DWA approach exhibit substantial deviations from the reference trajectory. The DWA approach exhibits a maximum variation of 1.547 m during the process of obstacle avoidance. Furthermore, the minimal divergence from the reference trajectory following successful obstacle avoidance is measured at 0.3303 m. Both of these processes exhibit noticeable instances of excessive and ineffective avoidance trajectories. On the other hand, the obstacle avoidance approach using NMPC demonstrates a maximum variation of 0.9035 m while navigating around obstacles. Furthermore, once the avoidance is successfully executed, it promptly converges towards the desired reference trajectory. During this particular phase, the largest divergence from the reference trajectory is recorded as 0.3183 m. This suggests that the obstacle avoidance method using NMPC is capable of efficiently navigating around obstacles within a narrower range of deviation, while simultaneously exhibiting a swift convergence towards the desired trajectory.

**Figure 12:**
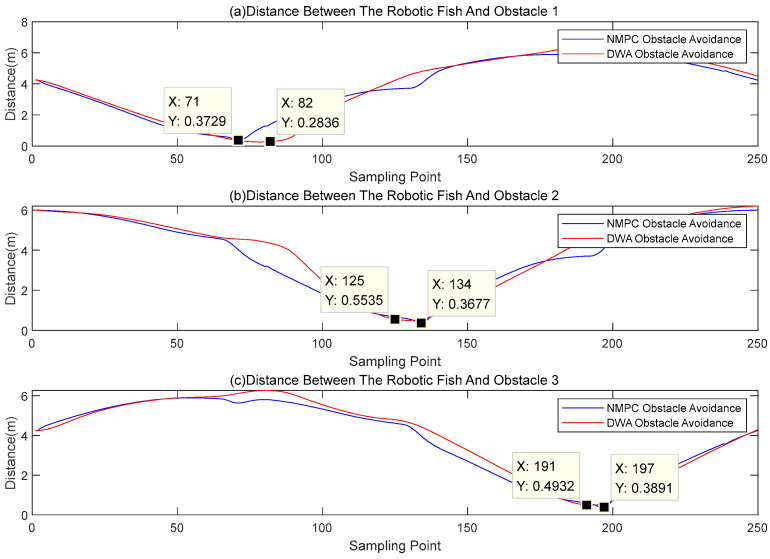
Distances between the centroid of the robotic fish and the centroids of obstacles.

In order to evaluate the efficacy of the DWA and NMPC obstacle avoidance techniques in safely maneuvering around obstacles, the distance between the centroid of the robotic fish and the centroids of the obstacles is employed as a metric. The findings are illustrated in Figure 12.

By examining Figure 12, it is observed that, when employing the DWA obstacle avoidance technique, the robot fish’s centroid maintains minimum distances of 0.2836 m, 0.5535 m, and 0.4932 m from the centroids of the three obstacles, respectively. The lowest distances associated with the three obstacles are 141.8%, 276.27%, and 246.6% of the respective obstacle radius, which is 0.2 m. The NMPC obstacle avoidance approach demonstrates control over the minimum distances, which measure 0.3729 m, 0.3677 m, and 0.3891 m. These distances correspond to 186.45%, 183.85%, and 194.55% of the obstacle radius, respectively. Based on the examination of the aforementioned data, it can be inferred that both approaches for obstacle avoidance consistently maintain a considerable margin of safety from the obstacles during the avoidance procedure. Moreover, within the framework of the NMPC obstacle avoidance approach, it has been observed that the minimum distance of the centroid exhibits a rather consistent behavior, hence preventing the occurrence of extremely large or short avoidance distances.

Based on the analysis of Figure 10, Figure 11 and Figure 12, it can be observed that the DWA obstacle avoidance method demonstrates adherence to safety standards. Nevertheless, it presents certain concerns, such as an excessive avoidance radius and a delayed convergence of the avoidance trajectory towards the reference trajectory. The obstacle avoidance mechanism employed by NMPC guarantees that both the process of avoidance and the resulting trajectory of the robot fish remain within acceptable ranges, so demonstrating its notable precision and reliability. Additionally, it can be observed that the minimum distances between the centroid of the robot fish and the centroids of the obstacles are all contained within a range of 1.5 to 2 times the radius of the obstacles. This finding serves to demonstrate the efficient and reasonably consistent obstacle avoidance capabilities of the NMPC approach.

## 5. Discussion

The exact control of robotic fish motion has long been a challenging task in the realm of underwater robotics, particularly inside complicated underwater settings. In order to tackle this particular difficulty, the present study utilizes a trajectory tracking and obstacle avoidance planning algorithm for autonomous underwater vehicles inspired by NMPC (Nonlinear Model Predictive Control). The method presented in this study aims to enhance control performance by incorporating real-time system status and external environmental information. This approach facilitates the achievement of agile and responsive control for the robotic fish. The initial step involves establishing the dynamic model of the robotic fish within the global coordinate system. Subsequently, the NMPC algorithm is delineated and accompanied by the formulation of obstacle avoidance and objective functions, hence facilitating the development of an NMPC controller that incorporates control restrictions.

**Figure 13:**
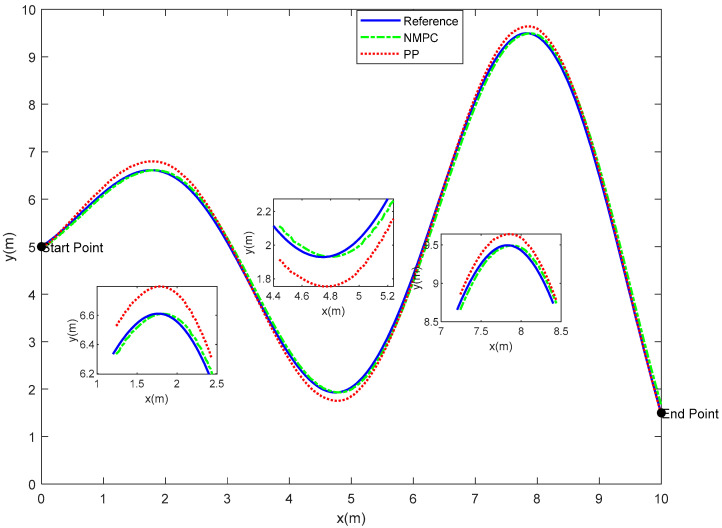
Trajectory Tracking Results of Robotic Fish on S-shaped Curve.

**Figure 14:**
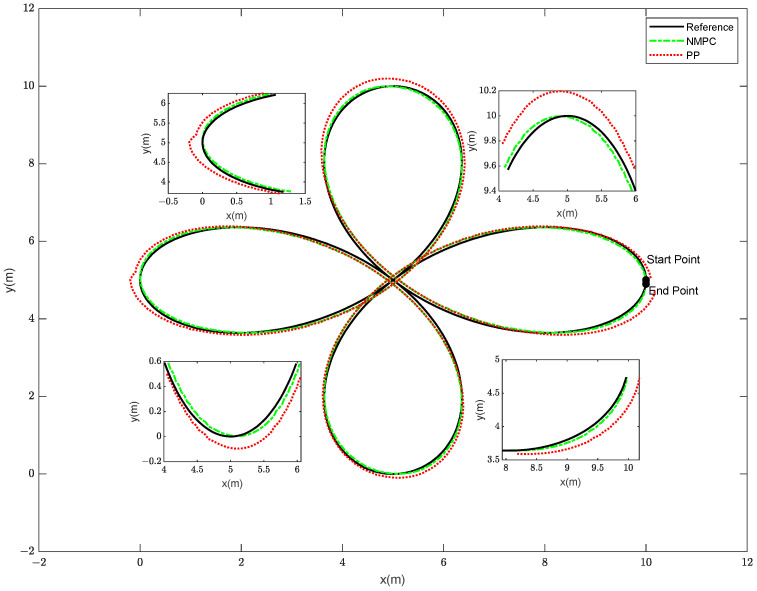
Trajectory Tracking Results of Robotic Fish on Four-leaf Clover Curve.

In order to assess the efficacy of this methodology, the NMPC algorithm and the PP algorithm were employed to achieve trajectory tracking on S-shaped and cloverleaf curves, respectively. The comparison results are shown in Figure 13 and Figure 14.

Both the NMPC tracking method and the PP tracking method have the capability to accurately follow the reference trajectory. In the case of straight or smooth pathways, both of these approaches provide comparable trajectories and demonstrate efficient control. Nevertheless, disparities in trajectory tracking between the Pure Pursuit (PP) and Nonlinear Model Predictive Control (NMPC) methods become apparent, particularly while navigating along S-shaped routes. In this scenario, the PP tracking approach exhibits a lower level of precision compared to NMPC. In contrast to the PP tracking approach, NMPC demonstrates superior performance in precisely tracking the reference trajectory on straight roads, as well as ensuring convergence to the reference trajectory on curved paths. The differences between the PP and NMPC tracking systems are apparent within the complex four-leaf clover curve environment. Premature turning may be observed in the trajectory of the PP tracking method at the extremities of the clover leaves, indicating a deviation from the reference path. On the other hand, it can be observed that the NMPC control approach exhibits a high level of precision in achieving convergence towards the desired trajectory at the leaf tips. This finding serves as empirical evidence supporting the efficacy of NMPC in accurately monitoring trajectories.

**Figure 15:**
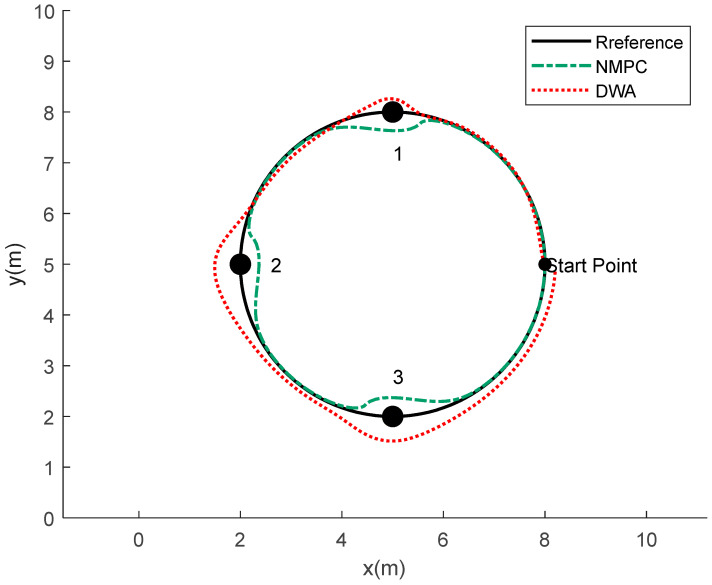
The planned trajectory for obstacle avoidance of the robotic fish.

Subsequently, a comparison examination was conducted to assess the obstacle avoidance planning capabilities of the NMPC algorithm and the DWA algorithm. The comparison results are shown in Figure 15.

The DWA obstacle avoidance approach satisfies the necessary conditions for ensuring safety. However, it does encounter several challenges, including an excessively large obstacle avoidance radius and a lack of rapid convergence of the avoidance trajectory to the intended path. On the other hand, the obstacle avoidance method using NMPC guarantees that the trajectory for avoiding obstacles stays within a suitable range during the collision avoidance procedure and after the avoidance maneuver is executed. This approach exhibits notable precision and stability. In addition, the obstacle avoidance method employed by NMPC ensures that there is a minimum distance maintained between the center of mass of the fish and the center of mass of the obstruction, which is within a range of 1.5–2 times the radius of the obstacle. This demonstrates the method’s commendable performance in terms of both effectively avoiding collisions and maintaining stability. This study examines the effectiveness of the NMPC algorithm in the context of obstacle avoidance planning, hence assessing its resilience.

The utilization of NMPC by the robotic fish in order to achieve trajectory tracking and plan for obstacle avoidance exhibits certain constraints, hence suggesting potential avenues for future enhancements.

### 5.1. Limitations

- One of the main challenges associated with NMPC approaches is their high computational cost, as they necessitate the recalculation of control algorithms at each time step. Therefore, in intricate underwater environments, particularly those that necessitate immediate reactions, the substantial computing complexity could potentially undermine real-time efficacy.
- The effectiveness of NMPC approaches is contingent upon the precision of the system dynamics models. Nevertheless, the dynamics of underwater environments can be impacted by various factors, including water flow and turbulence. The task of guaranteeing the precision of the model presents a formidable challenge, thus impacting the efficacy of NMPC.
- One limitation of NMPC methods is their inadequate adaptability to complex underwater environments. In situations characterized by extreme conditions or high complexity, these methods may struggle to effectively respond to various scenarios, particularly when faced with unfamiliar obstacles or rapidly changing conditions. Consequently, this can result in a decline in the performance of the NMPC algorithm.

### 5.2. Future Research Directions

- Future research endeavors may prioritize the refinement of NMPC algorithms, with a particular emphasis on augmenting computational efficiency, mitigating reliance on model correctness, and optimizing control parameters to effectively accommodate intricate underwater settings.
- Multi-Model Fusion: The integration of diverse sensor data and distinct control models is employed to raise the flexibility of the system to environmental variations, thus augmenting the system’s robustness.
- The proposed approach involves the integration of deep learning techniques with adaptive control methods, allowing the robotic fish to autonomously modify control strategies by leveraging learnt environmental patterns. This integration aims to enhance the adaptability of the robotic fish in complicated situations.

## 6. Conclusions

This study intends to achieve trajectory tracking and obstacle avoidance effects by enhancing the NMPC algorithm and implementing it on the nonlinear system of the robotic fish. Initially, the establishment of the global coordinate system and the body-fixed coordinate system, along with the transformation relationship between the body centroid of the fish and these coordinate systems, was undertaken. A kinematic model incorporating acceleration was formulated based on the provided information. This work presents a comprehensive examination of the NMPC approach, focusing on the formulation of obstacle avoidance functional functions and NMPC objective functions. In continuation of the previous discussion, a NMPC controller was developed, integrating control constraints and system constraints in order to guarantee the precision and stability of motion trajectories. The examination of the simulation outcomes revealed a close correspondence between the tracked trajectory of the centroid of the fish and the reference trajectory. When faced with barriers, the fish demonstrated the ability to successfully navigate around them, subsequently returning to the designated trajectory and ultimately reaching the intended destination. A comparative analysis between the PP tracking algorithm and the NMPC tracking algorithm revealed that the latter exhibited superior precision. In contrast to the DWA obstacle avoidance algorithm, the NMPC algorithm demonstrated superior levels of safety and stability. Nevertheless, it should be noted that the NMPC algorithm exhibited a comparatively higher computing burden and lengthier execution duration. Additional investigation is required to enhance and refine the NMPC algorithm in order to achieve optimal performance in the execution of simulation experiments. The primary objective of this study was to conduct a simulation with a solitary fish. Subsequent research endeavors may encompass exploring the concept of multi-fish formation control in order to attain trajectory tracking capabilities and effectively avoid obstacles.

## Data Availability

The datasets used or analysed during the current study are available from the corresponding author on reasonable request.
